# Exploiting Gene Expression Variation to Capture Gene-Environment Interactions for Disease

**DOI:** 10.3389/fgene.2012.00228

**Published:** 2013-05-31

**Authors:** Youssef Idaghdour, Philip Awadalla

**Affiliations:** ^1^Sainte-Justine Research Center, University of MontrealMontreal, QC, Canada

**Keywords:** eQTL, eSNP, gene-environment interactions, transcriptome

## Abstract

Gene-environment interactions have long been recognized as a fundamental concept in evolutionary, quantitative, and medical genetics. In the genomics era, study of how environment and genome interact to shape gene expression variation is relevant to understanding the genetic architecture of complex phenotypes. While genetic analysis of gene expression variation focused on main effects, little is known about the extent of interaction effects implicating regulatory variants and their consequences on transcriptional variation. Here we survey the current state of the concept of transcriptional gene-environment interactions and discuss its utility for mapping disease phenotypes in light of the insights gained from genome-wide association studies of gene expression.

## Introduction

**Figure 1:**
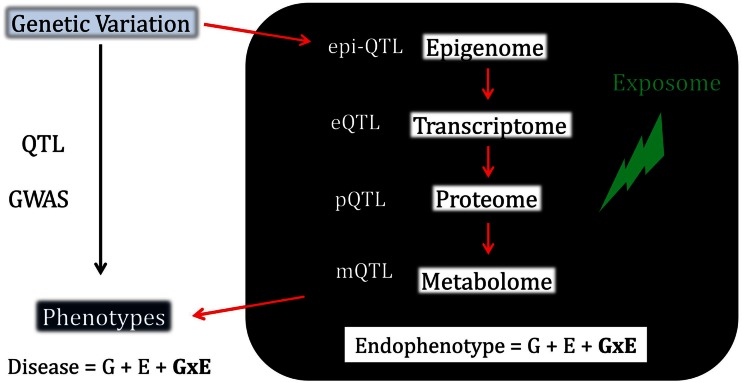
**To understand the etiology of disease, we have to understand how genotypic variation transduces into whole-organism phenotypic variation**. Gene-environment interactions have long been recognized as a fundamental component of this process. However, robust demonstrations of gene-environment effects in humans are scarce. Illuminating the mechanistic black box leading from genes to disease by characterizing variation at each – omics level and by capitalizing on the advantages and power gained from the use of endophenotypes holds the promise to address this major shortcoming in modern human biology.

The genotype-phenotype relationship is highly complex for most traits. Most of the complexity lies beneath higher-level phenotypes, moving from the architecture of the genome itself to protein function and transitioning through the complex modes of epigenetic, gene expression, and post-transcriptional regulation (Figure 1). Regulation of gene expression is the first stage in a multi-step process toward the production of phenotypes and is arguably the most important component in the genetic basis of phenotypic variation (Wray et al., 2003; Caroll, 2005). Transcript abundance sums the effects of various sources of variation in gene expression including genetic variation, spontaneous inherited epigenetics marks, and environmental factors. The latter include external stimuli and substances such as temperature, microorganisms, drugs, and chemicals. These causes of variation in gene expression can be variable at the cell, tissue, organism, or population level (Raser and O’Shea, 2005) and act together at various magnitudes on a battery of modulators that include promoters, activators, enhancers, repressors, *trans* effectors, chromatin, and environment- or genotype-dependent methylation state (Wray et al., 2003; Consortium et al., 2012).

## The Genetic Mapping of Genome-Wide Gene Expression

The concept of the genetic mapping of genome-wide gene expression takes advantage of the joint analysis of genotypic and gene expression data with the intention of gaining greater insight than can be provided by either type of data alone (Jansen and Nap, 2001; Morley et al., 2004; Stamatoyannopoulos, 2004; Gibson and Weir, 2005). This line of research was motivated by the basic idea that transcript abundance is a quantitative trait with a heritable component. Consequently, classical quantitative linkage mapping (QTL) methods can be used to map it to *cis*- and *trans*-acting sources of variation, also referred to as *local* and *distal* expression QTLs (eQTLs), respectively (Rockman and Kruglyak, 2006). eQTL mapping and gene expression heritability are therefore tightly linked (Dixon et al., 2007; Goring et al., 2007; Stranger et al., 2007; Skelly et al., 2009) and generally the term eQTL refers to a linkage signal from any types of loci (SNP, copy number variant, tandem repeats etc.) that are associated with transcript variation among relatives. The term expression single nucleotide polymorphism (eSNP) on the other hand denotes SNPs associated with variation in transcript abundance in a population of unrelated samples (Kim and Gibson, 2010).

Major advances in large-scale gene expression genotyping technologies have provided the opportunity to characterize thousands of eQTLs/eSNPs and to begin to understand their mechanisms of action. Arguably eQTLs and eSNPs can now be reproducibly identified by genome-wide screens in multiple populations and in various tissues (Cheung et al., 2005; Emilsson et al., 2008; Heinzen et al., 2008; Cookson et al., 2009; Dimas et al., 2009; Heap et al., 2009; Kwan et al., 2009; Idaghdour et al., 2010, 2012; Nica et al., 2011; Grundberg et al., 2012; Powell et al., 2012; Stranger et al., 2012). The large number of eQTL/eSNP studies published in the last few years is indicative of the increased interest in characterizing the genotypic modulators of gene expression. These studies have documented the presence of significant local, and to a much less extent distal, control of gene expression. However, distal effects are less tractable because of the complexity of transcriptional interactions and the caveat of multiple testing (Kim and Gibson, 2010).

## Transcriptional Gene-Environment Interactions

**Figure 2:**
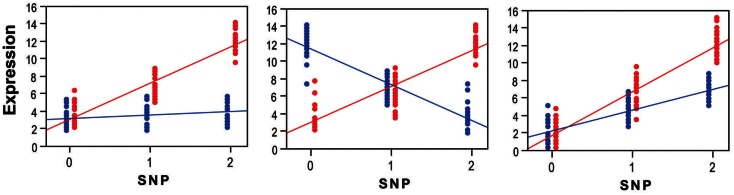
**Three main scenarios of transcriptional gene-environment interactions observed under two environmental conditions (e.g., exposed, red; vs. unexposed, blue) using standard linear regression, which is one of the most commonly used tests to detect interactions**. The panel to the left shows a scenario where the eSNP effect is observed only in the exposed group where the minor allele drives up expression levels. The major homozygote individuals show no differential expression between the exposed and unexposed conditions. The panel in the middle shows a scenario where the eSNP effect is present in both conditions but is in the opposite direction giving rise to significant differential expression in both homozygote classes. The panel to the right shows the scenario of a quantitative statistical interaction where the eSNP effect is observed in both conditions in the same direction but at different magnitudes. Genotypes on the *x*-axis are labeled to indicate the number of minor alleles and individuals are labeled to indicate their exposure status (exposed, red; unexposed, blue). On the *y*-axis are relative expression values.

Here we refer to gene-environment interactions as the differential effect of a given genotype exposed to different environmental conditions (Figure 2). These interactions can be *qualitative* (effects present only in one condition or going in opposite directions in different strata) or *quantitative* (effects go in the same direction but differ in magnitude). The presence and magnitude of quantitative interaction depends on several factors including the scale on which the phenotype is measured, how the “genotypic effect” is considered and the statistical approach adopted (Thompson, 1991; Falconer and MacKay, 1996; Kraft and Hunter, 2005; Dempfle et al., 2008; Thomas, 2010a,b). For example, in a linear regression of expression levels on a binary exposure, binary genotype, and a product gene-environment interaction term, the regression coefficient for the product interaction term represents the change across exposures in the difference in mean expression across genotypes. In a linear regression of log-transformed expression levels, the regression coefficient for the interaction term represents the change in the genotypic differences in mean log-transformed expression levels. The differences in mean expression levels are not generally the same as the differences in log-transformed levels. Hence, conclusions regarding the presence and magnitude of interaction effects depends on what precisely is meant by “effect,” which in turn usually depends on the scale used to define the outcome in a regression model.

Various statistical approaches including linear regression (to test for a linear trend, where heterozygotes are intermediate in phenotype owing to additive allelic effects, see Figure 2) and analysis of variance (ANOVA, to test for genotype effects irrespective of allelic trends) are used to decompose genetic and environmental effects into main and interaction effects (Thomas, 2010a,b; Yi, 2010). The range of phenotypes across a range of exposures resulting from a given genotype is referred to as a norm of reaction or phenotypic plasticity and constitutes an important feature in agricultural, genetic, evolutionary, as well as biomedical research. This phenomenon relates in theory to all organisms and virtually to all phenotypes. Quantitative traits in model organisms typically exhibit significant gene-environment interactions and have been reported for a range of organismal, behavioral, and biochemical phenotypes (MacKay, 2010). Increased knowledge about gene-environment interactions in humans has been gained through the implementation and integration of epidemiological and statistical approaches. These efforts gained momentum as large datasets became available and systematic surveys conducted, giving rise to a rapidly growing and often controversial literature on gene-environment interactions (Hunter, 2005; Munafo and Flint, 2009; Thomas, 2010a). Arguably, genuine cases of interaction effects for complex traits are the exception rather than the rule (Le Marchand et al., 2001; Wu et al., 2012), possibly due to a combination of poor study design, low power, data dredging, and failure to correct for multiple comparisons, as well as true heterogeneity in effects across the populations that have been studied. Furthermore, the pursuit of the goal of replication and functional validation of interactions, and ultimately making them biologically interpretable and clinically useful is undoubtedly challenging, particularly for complex traits (Matullo et al., 2005; Ioannidis, 2007; Thomas, 2010a). As a result, an appreciation for the need to address the challenges facing the quest to characterize gene-environment interactions in humans is rapidly gaining momentum (Bennett et al., 2011; Boffetta et al., 2012) and here we argue that taking advantage of endophenotypes, including transcript abundance, can provide added value to these efforts (Figure 1).

Transcript abundance, like any other quantitative trait, is the product of genotype, environment, and interaction effects. In contrast to the tremendous progress made in documenting main environmental and genotypic effects, systematic genome-wide surveys of transcriptional genotype-environment effects are scarce with the first notable findings reported for model organisms (Li et al., 2006; Landry et al., 2007; Sambandan et al., 2008; Smith and Kruglyak, 2008). These reports have emerged from experiments that provide a convenient framework to systematically survey interaction effects. Li et al. (2006) showed that differential expression induced at two different temperatures has a strong genetic component in *Caenorhabditis elegans* recombinant inbred strains derived from parental lines collected in the UK and Hawaii. This work documented that no less than 59% of 308 *trans*-acting and 8% of an estimated 188 *cis*-acting genes showed a significant eQTL-by-environment interaction. Landry et al. (2007) investigated whole-genome transcriptional plasticity of six yeast strains in four different conditions and reported 223 transcriptional strain-condition interaction effects for 5,258 genes assayed. Effects one order of magnitude stronger have been reported in the yeast where expression profiles of two strains in two different conditions were contrasted, showing evidence for 2,037 strain-condition interactions (Smith and Kruglyak, 2008). Further linkage analysis identified 1,555 eQTLs for 1,382 traits significant for the interaction term with *cis*-eQTLs shown to be more stable across conditions while *trans*-eQTLs were predominantly condition-dependent. On the other hand, a study investigating olfactory behavior in *Drosophila melanogaster* showed that 50% of variation in this trait was attributable to gene-environment interactions and reported 20 transcriptional gene-environment interactions albeit in whole flies and using a relaxed statistical significance threshold (Sambandan et al., 2008).

There is no *a priori* expectation for the number of transcriptional gene-environment interactions under the scenario of large gene-environment interaction effect on a given phenotype but this would depend largely on the genetic architecture of the phenotype in question. In an extreme example, if the trait depends heavily on expression of a single gene a gene-environment interaction at a single regulatory polymorphism with large effect on that gene could explain most of the gene-environment interaction effects on the trait. The notable success in detecting transcriptional gene-environment interactions in microorganisms is primarily a consequence of the tight link in time and space between transcript abundance and the environmental factors investigated. The contrasting findings on the extent of transcriptional gene-environment interactions that have been reported can be due to several reasons including the complexity of architecture of the genetic basis of the expression traits, species-specific patterns of gene expression regulation, and differences in the exposure under investigation.

## Transcriptional Gene-Environment Interactions in Humans

The literature on the topic of transcriptional gene-environment interactions in humans is slim with most of the comprehensive studies documenting genotype-treatment interactions (Table 1, reviewed also in Maranville et al., 2012). The first genome-wide survey of these effects was reported by Smirnov et al. (2009) who conducted a linkage study in human lymphoblast cell lines and found a predominance of *trans*-acting factors to be responsible for widespread transcriptional response to irradiation. Others reported dozens of interaction effects in response to pro-inflammatory oxidized phospholipids (Romanoski et al., 2010), glucocorticoids (Maranville et al., 2011), and *Mycobacterium tuberculosis* infection (Barreiro et al., 2012) arguing for genotype-treatment interactions in the cell type being investigated. These *in vitro* experiments where homogenous cell populations are investigated and transcriptional response deliberately altered provide increased power to detect interaction effects as effect sizes become more pronounced and readily detectable using modest sample sizes. Nonetheless these experiments rely heavily on prior knowledge about the cell type relevant to the treatment in question.

**Table 1:** **Recent published genome-wide surveys of transcriptional genotype-environment interactions**.

Exposure (Reference)	System – population	Sample size – analysis	Results
Radiation (Smirnov et al., 2009)	Lymphoblastoid cell lines – European (HapMap CEPH)	15 (baseline, 2 h, and 6 h post irradiation) – linkage analysis, 4,600 SNPs, significance threshold: *p* = 0.05 experiment-wide	>1,200 radiation-induced expression traits show significant linkage to specific chromosomal regions
Geography/lifestyle (Idaghdour et al., 2010)	Leukocytes – Morocco (North African ancestry)	194 (rural vs. urban lifestyle) – Linear regression, >500 k SNPs, significance threshold: *p* = 0.05 experiment-wide	Genotype and environment act largely in an additive manner with suggestive evidence of subtle interaction effects
Oxidized phospholipids (Romanoski et al., 2010)	Primary aortic endothelial cells – largely European with some Asian and African ancestries	96 (with and without treatment) – Linear regression, >500 k SNPs, 5% FDR	18 genes show evidence for an interaction between an eSNP and oxidized phospholipid treatment
Synthetic glucocorticoid dexamethasone (Maranville et al., 2011)	Lymphoblastoid cell lines – African and European (HapMap)	114 (with and without treatment) – Bayesian regression, >500 k SNPs, significance threshold: 10% FDR	26 genes show evidence for an interaction between an eSNP and dexamethasone treatment
*Mycobacterium tuberculosis* (Barreiro et al., 2012)	Primary dendritic cells – Caucasian	65 – (infected and non-infected) – linear regression, >800 k SNPs, significance threshold: eSNP at 1% FDR only in one condition and no signal in the other condition at 50% FDR	198 genes show evidence for an interaction between an eSNP and MTB infection
*Plasmodium falciparum* (Idaghdour et al., 2012)	Whole blood – West Africans	151 – (infected and non-infected) – linear regression, >500 k SNPs, significance threshold: *p* = 0.05 experiment-wide	5 genes are subject to genome-wide significant transcriptional genotype-infection interaction effects and dozens of eSNP associations are sensitive to infection

Interaction effects can also be investigated in tissues with a heterogeneous cell composition but robust statistical analysis is required to tease apart true effects from those reflecting differential cell proportions between the groups contrasted (Emilsson et al., 2008; Stegle et al., 2010). For example, the whole leukocyte fraction of peripheral blood is a readily available system that does not require further sample manipulation and can be useful for the discovery of interaction effects *in vivo*. However the system is made up of over a dozen cell types and overall profile differences are due to the joint contributions of variable cell fractions, and variable expression within cells. For instance, Fairfax et al. (2012) characterized eSNPs in B lymphocytes and peripheral blood monocytes from the same set of individuals and demonstrated that nearly 80% of eSNPs were identified in only one of the two cell types with 31 genes showing significant opposite directional eSNP effects in the two cell types, differences which would be missed when studying total peripheral blood cells. Nevertheless experiments on whole blood provide general insights that are useful to generate specific hypotheses and help design follow up experiments to further characterize putative interactions in specific cell types. Genome-wide surveys of transcriptional gene-environment interactions *in vivo* in human transcriptomes have also been conducted. A study investigated differences in the pattern and strength of genotype-expression association in fresh human transcriptomes across groups sampled from different geographic locations suggested the presence of several interaction effects beneath genome-wide significance but no genome-wide significant interactions were reported likely due to lack of power (Idaghdour et al., 2010). Another report surveyed host genotype-infection interactions in whole blood in response to *Plasmodium falciparum* infection (Idaghdour et al., 2012) and reported five genes for which the eSNP effect is highly dependent on infection status translating into genome-wide significant interactions. Dozens of other interactions beneath genome-wide significance were reported as well.

It is worth noting that studies mapping interaction effects vary widely in their methodologies, statistical approach, and the significance threshold applied to call interactions and there is a pressing need for a clear formulation of the concept of “transcriptional gene-environment interaction” so that published findings can be reliably contrasted. The issue of dependence of the definition of interaction on the scale at which the trait is measured is of particular importance here, given that the first phase of the analysis of gene expression datasets involves normalization that can influence biological inference as evidenced by a systematic evaluation of the effect of nine different normalization strategies on various aspects of statistical inference including eSNP analysis (Qin et al., 2012). This said, the few published reports on transcriptional gene-environment interactions all show how in principle an individual’s response to an environmental perturbation can be moderated by his or her genetic make-up through modulation of gene expression with likely consequences on disease physiology and disease susceptibility.

## Mapping Power and Genotype-Phenotype Mapping

A major challenge for GWAS in general is that of power and the problem of multiple testing. This caveat is more pronounced for the discovery of interaction effects particularly when testing interactions with multiple environmental factors and given the high dimensionality of the data and the fact that in general detecting an interaction requires at least a fourfold larger sample size than does a main effect of comparable magnitude (Smith and Day, 1984; Luan et al., 2001). For instance, in a simple linear design where the interaction effect is tested for one environmental exposure (e.g., treatment vs. no-treatment) and one SNP with three genotypes (e.g., AA; AG and GG), there are six genotype-environment combinations that are handled simultaneously, thus requiring an appropriate sample size for each combination to ensure power. This issue is more pronounced when effect sizes of genotype are modest and allelic frequencies are intermediate or low (Ege et al., 2011), and particularly in the presence of heterogeneity in the sample whether it is genetic (e.g., genetic background, ethnicity) or environmental (e.g., differential exposures, environmental residual error). Discovery of transcriptional gene-environment interactions suffer less from these issues. Transcript abundance is a continuous trait, hence potentially more informative and provides more mapping power, and it is closer to the genetic effect and/or the causal mechanisms of exposure. Genotypes have effects on transcript abundance on average one order of magnitude stronger than on disease phenotypes reflecting the tight link between genetic regulatory elements and gene expression traits. Typically in a sample as small as 100 individuals, a few hundred peak eSNPs are detected explaining on average a third of the variance of the transcript abundance of the associated gene (Kim and Gibson, 2010).

We argue that efforts to detect genuine gene-environment interactions in humans are more likely to be fruitful when using integrative approaches that capitalize on the advantages and power gained from the use of endophenotypes. These efforts will certainly be enhanced as more cohort studies and population-based biobanks turn their attention to deep endophenotyping (Awadalla et al., 2012) and profiling of multiple tissues and cell types (Dermitzakis, 2012; Grundberg et al., 2012) that facilitate the investigation of multiple levels of – omics data. Our own efforts are currently focused in this direction as we are performing comprehensive genomic profiling using next-generation approaches that include deep RNA sequencing of samples collected from 20,000 deeply endophenotyped individuals from the only population-based health survey and biobank in QC, Canada – the CARTaGENE project (Awadalla et al., 2012). Twenty-thousand participants is a moderately sized cohort, but with integrative technologies and approaches, there is substantial power to capture both eSNPs associated with endophenotypes, and heterogeneity in profiles across geographic regions. Other initiatives are taking similar approaches (e.g., the MuTHER, PROOF, and Framingham projects) warranting the need for harmonizing collection protocols for transcriptomic data as was the case with phenotypic and genetic variation data (McCarthy et al., 2008; Manolio et al., 2009; Bennett et al., 2011).

Knowledge of gene-environment interactions in humans holds the promise to enhance our understanding of the etiology of disease but also highlights the need to address several issues and questions related to this phenomenon. For example, the relationship between gene-environment interactions at the transcriptional level and at the disease level is a subject that warrants investigation. Unraveling these relationships in humans will be challenging given the extent of biological complexity of the black box between genotype and phenotype. Presumably transcriptional changes might be silent and do not result in phenotypic change. Distinct transcription profiles might also converge and yield similar phenotypes as well as the possibility that mechanisms of post-transcriptional regulation may account for buffering transcriptional variation and therefore break the otherwise statistically significant associations between gene expression levels and higher-level phenotypes. Furthermore, the generality of transcriptional genotype-environment interactions across multiple loci, particularly those involved in the etiology of disease, would cause a major hurdle for the identification of disease variants and might account for some of the failures of replication of associations where main genotype effects are estimated over a constellation of conditions represented in a study as is the case in GWAS.

## Conclusion

In conclusion, the insight that is coming out of the fields of quantitative genetics and epidemiology is the appreciation of the role gene-environmental interactions play in shaping disease phenotypes. However, robust demonstrations of these effects in humans are scarce. Genome-wide association studies of gene expression in various tissues and populations have opened up the opportunity to track and discover these effects in the transcriptome and to shift from focusing solely on main genotypic effects. The evidence in the literature of the prevalence of such effects in the human transcriptome is increasing, but these studies are still in their infancy and need to be extended to scenarios where regulatory variation is expected to depend on environmental triggers. It will also be interesting to explore and expand on these studies to see if the epigenome is subject to interaction effects (Cortessis et al., 2012). This is particularly relevant in the context of mapping the genetic basis of disease susceptibility and personalized medicine.

## Conflict of Interest Statement

The authors declare that the research was conducted in the absence of any commercial or financial relationships that could be construed as a potential conflict of interest.
